# Initial clinical trial of epratuzumab (humanized anti-CD22 antibody) for immunotherapy of systemic lupus erythematosus

**DOI:** 10.1186/ar1942

**Published:** 2006-04-21

**Authors:** Thomas Dörner, Joerg Kaufmann, William A Wegener, Nick Teoh, David M Goldenberg, Gerd R Burmester

**Affiliations:** 1 Department of Medicine/Rheumatology and Clinical Immunology, Charite Hospital, Berlin, Germany; 2 Immunomedics, Inc., Morris Plains, NJ, USA; 3 Center for Molecular Medicine and Immunology, Belleville, NJ, USA

## Abstract

B cells play an important role in the pathogenesis of systemic lupus erythematosus (SLE), so the safety and activity of anti-B cell immunotherapy with the humanized anti-CD22 antibody epratuzumab was evaluated in SLE patients. An open-label, single-center study of 14 patients with moderately active SLE (total British Isles Lupus Assessment Group (BILAG) score 6 to 12) was conducted. Patients received 360 mg/m^2 ^epratuzumab intravenously every 2 weeks for 4 doses with analgesic/antihistamine premedication (but no steroids) prior to each dose. Evaluations at 6, 10, 18 and 32 weeks (6 months post-treatment) follow-up included safety, SLE activity (BILAG score), blood levels of epratuzumab, B and T cells, immunoglobulins, and human anti-epratuzumab antibody (HAHA) titers. Total BILAG scores decreased by ≥ 50% in all 14 patients at some point during the study (including 77% with a ≥ 50% decrease at 6 weeks), with 92% having decreases of various amounts continuing to at least 18 weeks (where 38% showed a ≥ 50% decrease). Almost all patients (93%) experienced improvements in at least one BILAG B- or C-level disease activity at 6, 10 and 18 weeks. Additionally, 3 patients with multiple BILAG B involvement at baseline had completely resolved all B-level disease activities by 18 weeks. Epratuzumab was well tolerated, with a median infusion time of 32 minutes. Drug serum levels were measurable for at least 4 weeks post-treatment and detectable in most samples at 18 weeks. B cell levels decreased by an average of 35% at 18 weeks and remained depressed at 6 months post-treatment. Changes in routine safety laboratory tests were infrequent and without any consistent pattern, and there was no evidence of immunogenicity or significant changes in T cells, immunoglobulins, or autoantibody levels. In patients with mild to moderate active lupus, 360 mg/m^2 ^epratuzumab was well tolerated, with evidence of clinical improvement after the first infusion and durable clinical benefit across most body systems. As such, multicenter controlled studies are being conducted in broader patient populations.

## Introduction

Systemic lupus erythematosus (SLE) is a prototypic autoimmune disease that can involve many organ systems [1]. In Europe and the United States, estimates of the number of affected individuals range from 24 to 65 cases per 100,000 people [1,2]. The clinical course of SLE is episodic, with recurring activity flares causing increasing disability and organ damage. Cyclophosphamide, azathoprine, and corticosteroids remain important for long-term management of most patients having active disease, and even those in clinical remission [1]. Despite the important advances made with these drugs, especially cyclophosphamide, in controlling lupus disease activity, they have considerable cytotoxicity and cause, for example, bone marrow depression, ovarian failure, enhanced risk of bladder cancer, as well as the known side effects of long-term systemic corticosteroid therapy. As such, there continues to be a need for the development of targeted and less toxic therapies.

Specific autoantibodies against nuclear, cytoplasmic, and membrane antigens remain the serological hallmark of SLE. While lymphopenia is common, there is an increase in the level of activated B cells [3,4] and characteristic alterations of B cell subpopulations [5,6] that may be driven by extrinsic or intrinsic factors. B cells appear to have a key role in the activation of the immune system, in particular through the production of cytokines and by serving as antigen-presenting cells (reviewed recently in [7] ). Although B cell activation can occur independently of T cell help in lupus, a substantial fraction of B cells is activated in a T cell dependent manner [8-10], as demonstrated by isotype switching and affinity maturation of B cells [11,12] and enhanced CD154-CD40 interactions [13]. Useful insight into the pathogenesis of lupus has been obtained with animal models. MRL/lpr mice spontaneously develop a lupus-like autoimmune disease in an age-dependent manner, including autoantibody production, arthritis, skin lesions, and severe nephritis, which usually leads to early demise from renal failure [14]. When rendered B cell deficient, they no longer develop nephritis, mononuclear infiltrates are no longer detectable in the kidneys or skin, the number of activated memory T cells are markedly reduced, and infusions of pooled serum from diseased MRL/lpr mice lead to glomerular antibody deposition, but not the development of renal disease [15,16]. However, when reconstituted with B cells not able to secrete circulating antibodies, they develop nephritis and vasculitis [17]. As such, it appears that B cells play a direct role in promoting disease beyond the production of autoantibodies [18].

Depleting B cells with anti-CD20 monoclonal antibodies has emerged as a potentially new therapeutic strategy for certain autoimmune diseases. The chimeric monoclonal antibody rituximab depletes B cells by targeting the pan-B cell surface antigen CD20. Preliminary experience with rituximab in about 100 patients with SLE (recently reviewed in [7] ) and other autoimmune diseases has been encouraging [6,19-22].

Due to the central role of B cells in the pathogenesis of certain autoimmune diseases, targeted anti-B cell immunotherapies would be expected to offer therapeutic value in the setting of SLE. In addition to CD20, another unique target is CD22, a 135 kDa glycoprotein that is a B-lymphocyte-restricted member of the immunoglobulin superfamily, and a member of the sialoadhesin family of adhesion molecules that regulate B cell activation and interaction with T cells [23-27]. CD22 has seven extracellular domains and is rapidly internalized when cross-linked with its natural ligand, producing a potent co-stimulatory signal in primary B cells [25,28-30]. The function of CD22 in cell signaling is suggested by six tyrosine and three inhibitory domain sequences in the intra-cellular cytoplasmic tail. These inhibitory domains are phosphorylated by the non-receptor kinase Lyn upon B cell antigen receptor (BCR) activation by IgM ligation, leading to the activation and recruitment of SHP-1 phosphatase [31,32]. SHP-1 is a tyrosine phosphatase that negatively regulates several intracellular signaling pathways, including the calcium pathway, through dephosphorylation of signaling intermediates, such as Lyn and Syk. CD22 is first expressed in the cytoplasm of pro-B and pre-B cells, and then on the surface of B cells as they mature, with expression ceasing with B cell differentiation into plasma cells [23]. Studies in CD22-deficient mice and in CD22-negative cell lines have shown an increase in calcium response to BCR ligation [33-36], indicating that CD22 inhibition of BCR signaling is achieved through the mechanism of controlling calcium efflux in B cells. It has been reported that this effect of CD22 is mediated by potentiation of plasma membrane calcium-ATPase and requires SHP-1 [37]. Animal experiments indicate that CD22 plays a key role in B cell development and survival, with CD22-deficient mice having reduced numbers of mature B cells in the bone marrow and circulation, and with the B cells also having a shorter life span and enhanced apoptosis [31].

Therefore, CD22 is an attractive molecular target for therapy because of its restricted expression; it is not exposed on embryonic stem or pre-B cells, nor is it normally shed from the surface of antigen-bearing cells. Initially, a mouse monoclonal antibody (mLL2, formerly EPB-2) was developed and characterized that specifically binds to the third domain of CD22 [38,39]. Immunohistological evaluation revealed that it recognized B cells within the spleen and lymph nodes, but did not react with antigen unrelated to B cells in normal and solid tumor tissue specimens, and flow cytometry showed no reactivity with platelets, red blood cells, monocytes, and granulocytes in normal peripheral blood [38,39]. The complementarity-determining regions of mLL2 were subsequently grafted onto a human IgG_1 _genetic backbone [40]. Epratuzumab, the resulting complementarity-determining region-grafted (recombinant) 'humanized' monoclonal antibody (hLL2), is 90% to 95% of human origin, thus greatly reducing the potential for immunogenicity. Epratuzumab has been shown to mediate antibody-dependent cellular cytotoxicity *in vitro*[41] , and may also exhibit biological activity through modulating BCR function (J Carnahan, R Stein, Z Qu, K Hess, A Cesano, HJ Hansen, DM Goldenberg, manuscript submitted).

In clinical trials, over 400 patients with non-Hodgkin lymphoma (NHL) or other B cell malignancies have received epratuzumab administered as 4 consecutive weekly infusions over about 60 minutes. An initial phase I/II study administered doses of up to 1,000 mg/m^2^, with patients premedicated each week with oral acetaminophen and diphenhydramine to minimize potential infusion reactions. Epratuzumab toxicity consisted primarily of mild to moderate transient infusion-related events during the first infusion, and only one patient with a prior right lung resection for a fungal abscess had a serious event (bronchospasm during infusion), which was treated with parenteral medications. Based on this safety record, objective evidence of tumor response, and less severe depression of circulating B cells [42,43] , 4 consecutive weekly doses of 360 mg/m^2 ^epratuzumab was selected as a sufficiently safe and efficacious treatment regimen to warrant further clinical development. A pharmacokinetic analysis of weekly dosing subsequently demonstrated that the post-treatment serum half-life of epratuzumab in NHL patients was 19 to 25 days, consistent with the half-life of a human IgG_1 _[44]. As such, a longer interval between doses was indicated, and a biweekly dosing schedule was selected for this initial study in SLE. We report here the first experience of treating an autoimmune disease with a CD22 antibody, epratuzumab.

## Materials and methods

This initial, phase II, open-label, non-randomized, single-center study was undertaken to obtain preliminary evidence of therapeutic activity in SLE, to confirm the safety, tolerance and lack of immunogenicity of epratuzumab in this population, and to evaluate pharmacokinetic and pharmacodynamic parameters. The study was approved by the Ethics Committee of Charité University Hospital.

### Patient population

Males or non-pregnant, non-lactating females, ≥ 18 years of age, were eligible to participate provided they had a diagnosis of SLE according to the American College of Rheumatology revised criteria (fulfilled ≥ 4 criteria), with SLE for at least 6 months, and at least one elevated autoantibody level (antinuclear antibodies/ANA and/or anti-dsDNA) and moderately active disease (a score of 6 to 12 for total British Isles Lupus Assessment Group (BILAG) disease activity) at study entry. Patients were excluded if they had prior rituximab or other antibody therapy, allergies to murine or human antibodies, experimental therapy within 3 months, active severe CNS (central nervous system) lupus, laboratory abnormalities (hemoglobin < 8.0 g/dl, WBC (white blood cells) < 2,000/mm^3^, ANC (absolute neutrophil cells) < 1,500/mm^3^, platelets < 50,000/μl, liver transaminases or alkaline phosphatase more than twice upper limit of normal, serum creatinine > 2.5 mg/dl, or proteinuria > 3.5 gm/day), thrombosis, drug or alcohol abuse, infection requiring hospitalization within 3 months, long-term active infectious diseases (tuberculosis, fungal infections) within 2 years, malignancy (except basal cell carcinoma, cervical carcinoma in situ (CIS), history of recurrent abortions (2 or more), or known HIV, hepatitis B or C, or other immunosuppressive states.

### Concomitant medications

Pulsed methylprednisolone, other high-dose corticosteroids, cyclophosphamide, and intravenous, joint, or intramuscular corticosteroid injections were not allowed during the study or within four weeks of study entry. Low-dose corticosteroids (prednisone, = 20 mg/day or equivalent) or background therapy with standard antirheumatic immunosuppressives (for example, azathioprine, methotrexate) was permitted provided there were no dosing changes during the study or within four weeks prior to study entry. Antimalarials, non-steroidal anti-inflammatory drugs (NSAIDs), ACE-inhibitors or angiotensin receptor antagonists were also allowed, provided there were no dosing changes during the study or within two weeks of study entry.

### Treatment schedule

After satisfying eligibility, signing informed consent, and undergoing baseline evaluations, all patients received 4 doses of 360 mg/m^2 ^epratuzumab administered every other week with paracetamol (acetaminophen) and an antihistamine (but no steroids) given as premedication prior to each dose.

### Study evaluations

The BILAG system was used to categorize the severity level of lupus disease activity in each patient at study entry and at post-treatment evaluations obtained at 6 (24 hours after the last infusion), 10 and 18 weeks and at an additional 32 weeks (6 month post-treatment) follow-up visit. The BILAG system organizes lupus-associated signs and symptoms according to eight body systems: general/constitutional, mucocutaneous, neurological, musculoskeletal, cardiovascular/respiratory, vasculitic, renal, hematological domains [45,46]. At each evaluation, the presence and change of any signs and symptoms were recorded and the level of any disease activity within each body system determined on a treatment-intent basis, according to BILAG rules as: A (severely active disease sufficient to require disease-modifying treatment, for example, > 20 mg/d prednisolone, immunosuppressants/cytoxics); B (moderately active disease requiring only symptomatic therapy, for example < 20 mg/d prednisolone, antimalarials, NSAIDs alone or in combination); or C (stable mild disease with no indication for changes in treatment). To assign an overall disease activity level for each patient, a total BILAG score was determined by adding a numerical severity score (A = 9, B = 3, C = 1, no activity = 0) across the eight body systems. Other evaluations at these times included an SLE panel (autoantibodies, C3, C-reactive protein/CRP, erythrocyte sedimentation rate/ESR, other laboratory tests), vital signs, physical examination, adverse events, routine safety laboratory tests (hematology, serum chemistry), urinalysis, serum immunoglobulins, peripheral blood B and T cells, epratuzumab serum levels (analyzed by sponsor), and human anti-human (epratuzumab) antibody titers (HAHA; analyzed by sponsor).

### Human anti-human (epratuzumab) antibody assay

The sponsor's HAHA test is a competitive ELISA assay, where the capture reagent is epratuzumab and the probe is an anti-epratuzumab-idiotype antibody. The anti-idiotype antibody is an acceptable surrogate for what is reacted against in an immunogenic response by humans against the binding portion of epratuzumab that distinguishes the molecule from other human antibodies (for instance, the framework region that has human amino acid sequences). Test results are derived from an eight-point standard curve with varying dilutions of anti-idiotype antibody in bovine serum albumin. Patient serum samples are diluted 1:2 with bovine serum albumin and assayed in triplicate. The anti-idiotype standard curve is used to determine the presence of HAHA in unknown samples. An acceptable assay is based on linear regression parameters that must be met to define a valid assay.

### Statistical analyses

The primary assessment of disease activity compared post-treatment BILAG results with those at study entry, using total BILAG scores for overall assessment and letter grade categories to assess the level of disease activity within each body system. Adverse events and safety laboratory tests were graded according to NCI CTC version 3.0 criteria on a 1 to 4 scale for toxicity (1, mild; 2, moderate; 3, severe; 4, life threatening). All analyses of efficacy, safety, tolerance, immunogenicity, pharmacokinetics, and pharmacodynamics used descriptive statistics. Wilcoxon signed rank test was used to assess the statistical significance of changes in total BILAG scores compared to their baseline values. All statistical tests used a significance level of 0.05.

## Results

### Demographics and patient characteristics at study entry

A total of 14 Caucasian patients (13 females and 1 male; 23 to 53 years old, median age 40 years) were enrolled. At study entry, the patients had been initially diagnosed with SLE 1 to 19 years (median 10 years) earlier and were receiving corticosteroids (*n *= 13, 1 to 12 mg/day prednisolone) plus immunosuppressives (*n *= 11, including 50 to 200 mg/day azathioprine, *n *= 9; 20 mg/day methotrexate, *n *= 2; 2 g/day mycophenalate mofetil, *n *= 1), and antimalarials (*n *= 6, 200 to 600 mg/day hydroxychloroquine). All patients had positive ANA at study entry (titers of 80:1 to 5,120:1), and 5 patients (36%) had positive anti-dsDNA antibody levels (> 10 U/ml). Ten patients (71%) had ESR values that were elevated (> 15 mm/h) and 4 patients (29%) had raised CRP levels (> 0.5 mg/dl), while only 3 patients (21%) had C3 levels that were borderline low or decreased (< 90 mg/dl), and no patient had positive direct Coombs' or serum haptoglobulin levels elevated above borderline.

All patients had total BILAG scores of 6 to 12 (median 10) at study entry. No patient had A-level disease activity in any body system, 13 patients had B-level disease activity in at least one body system (2 with three Bs, 9 with 2 Bs, 2 with one B) and one patient had only C-level activities. B-level disease occurred primarily in the mucocutaneous, vasculitis, and general/constitutional body systems, with no B-level disease activity in the neurological or renal systems (Table 1), while C-level disease occurred primarily in the general/constitutional, musculoskeletal, hematological and neurological body systems (Table 2). The actual signs and symptoms at study entry that contributed to the B-level disease activity according to the BILAG rules are also summarized in Table 1, while those contributing to C-level disease activity are summarized in Table 2.

### Study drug administration

Twelve of the 14 patients (86%) completed all 4 infusions of 360 mg/m^2 ^epratuzumab as scheduled, while one patient with sleepiness attributed to premedication IV antihistamines prematurely terminated the first infusion but subsequently completed all 3 remaining infusions without further event, and one patient completed the first two infusions, but discontinued further infusions after development of herpes zoster, which responded to antivirals. The infusions were well tolerated, with a median infusion time of 32 minutes (23 to 86 minutes), and with infusion reactions in 6 patients all limited to occurrences of transient, mild (grade 1 NCI toxicity) adverse events (flu-like symptoms, tracheitis/throat ache, *n *= 2; arthralgia/myalgia, fever, fatigue, nausea, headache, chills, or rash, *n *= 1).

### Post-treatment evaluations and follow-up

All patients remained in the study through the 18-week post-treatment evaluation period. One patient had a late 18-week visit that fell within the 32-week time frame and the corresponding data were hence re-assigned to the 32-week visit. The single patient who did not complete all 4 infusions continued to receive post-treatment evaluations beginning at the 10-weeks follow-up visit. Except for the aforementioned deviations, all patients received post-treatment evaluations at 6, 10, and 18 weeks. One patient was lost to follow-up after 18 weeks, while 13 patients returned for the final 32-week evaluations (8 patients as scheduled, 5 with a delayed visit between 42 to 82 weeks).

### BILAG treatment response

**Figure 1:**
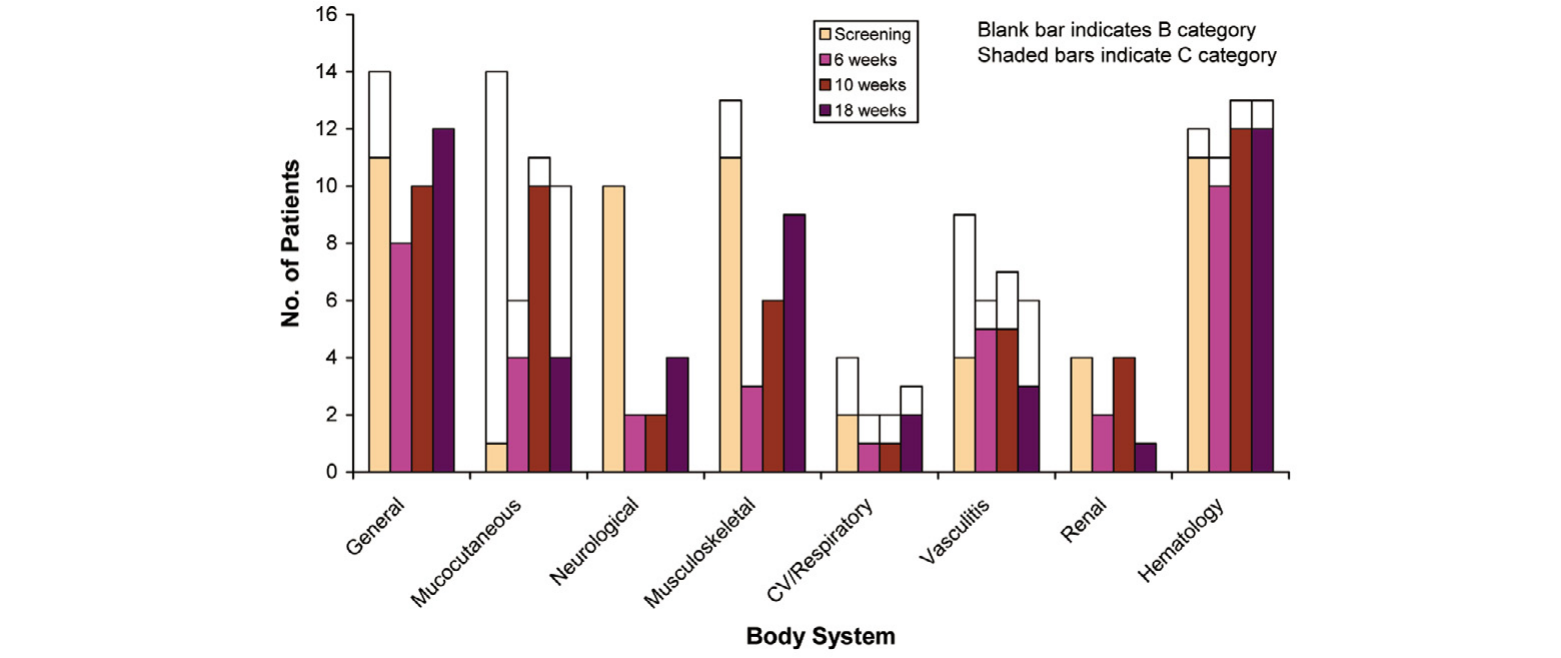
Frequency comparison of BILAG B- and C-level activities for each body system at screening, 6, 10 and 18 weeks.

**Figure 2:**
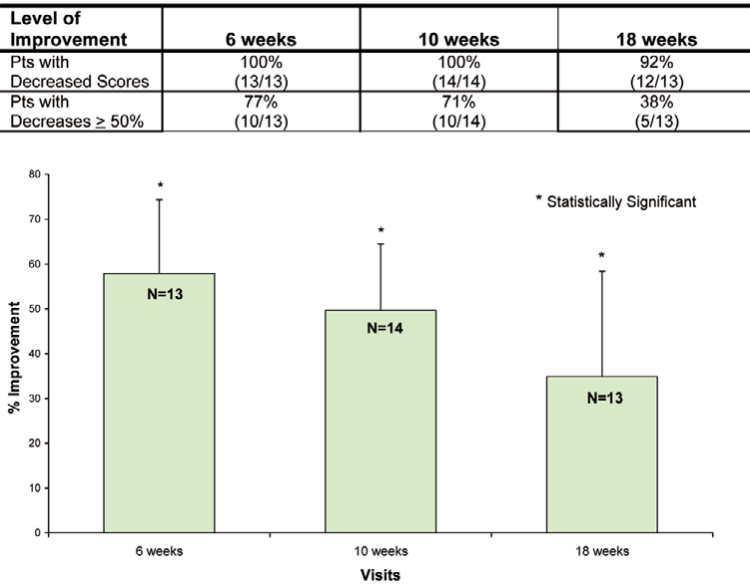
Overall frequency and mean improvement of total disease activity as measured by the total BILAG score at 6, 10 and 18 weeks.

**Figure 3:**
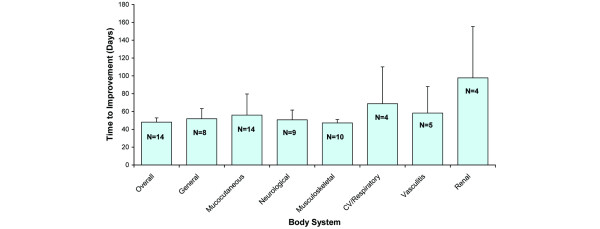
Mean time to improvement of each BILAG body stystem. Mean time to improvement (in days) of each BILAG body system during the follow-up of the study (N denotes the number of patients available for analysis for each body system). Since the first evaluation was scheduled for 6 weeks, the earliest time to improvement is at least 42 days.

The effect of epratuzumab on clinical manifestations was evaluated at 6, 10, and 18 weeks using numerical total BILAG scores as well as categorical scores. The compositions of B- and C-level activities improved after treatment, primarily in the general, mucocutaneous and musculoskeletal systems (Figure 1). Improvement in C-level activity was also observed in the neurological and renal domains. Improvements in the general, mucocutaneous, neurological and musculoskeletal systems occurred earlier compared to the cardiovascular/respiratory, vasculitic and renal systems (Figure 2). However, the limited number of patients with manifestations in each of these systems precludes a definitive determination of preferential effects. In terms of changes in the total BILAG score, statistically significant improvement was observed at 6, 10, and 18 weeks (Figure 3). Additionally, a substantial proportion of patients showed 50% or more improvement in total BILAG score at weeks 6, 10, and 18 (77%, 71% and 38%, respectively). At the final 32-week evaluation, statistically significant improvement in total BILAG score continued to be observed, with 15% of the patients achieving 50% or more improvement.

In a separate analysis, the total number of patients who achieved BILAG improvements in the particular domains at 6, 10 and 18 weeks of follow-up are summarized in Table 3. This indicates that the most characteristic BILAG domains, as also seen in Figure 2, were more likely to respond, although the duration of response was very similar throughout the domains. In fact, deterioration in BILAG categorical scores compared to baseline was infrequently seen during the study (Table 4). Only two patients (14%) showed worsening of hematological parameters (lymphocytopenia), one starting at 6 weeks and the other at 18 weeks. Another patient manifested renal (mild proteinuria) deterioration at 10 weeks. Overall, at week 18, 3 patients (21%) had a deteriorated BILAG assessment in at least one body system compared to baseline.

An additional analysis was performed to determine the durability of resolution of certain B- and C-level activities (Table 5). Although in a number of patients, B- and C-level activities resolved persistently, the heterogeneity of patients' manifestations again precluded the identification of a preferential response profile to the drug.

### Safety

During or following treatment, a total of ten patients reported adverse events. As reported above, six had mild, transient, infusional reactions and one patient experienced somnolence following antihistamine medication. Subsequently, five patients had infections (including herpes zoster, otitis media, *Helicobacter pylori-*associated gastritis, vaginitis/vaginal candidiasis, cystitis, and tonsillitis) that resolved with appropriate treatment, and one patient had spinal contusion from a traffic accident. Standard safety laboratory tests showed no consistent pattern of change from baseline, and infrequent post-treatment increases in NCI CTC v3.0 toxicity grades for these laboratory tests were all limited to changes of one grade level except for one patient with an increase in lymphocytes from grade 1 to grade 3, and another from grade 0 to grade 3 (Table 6).

### Pharmacokinetics and immunogenicity

**Figure 4:**
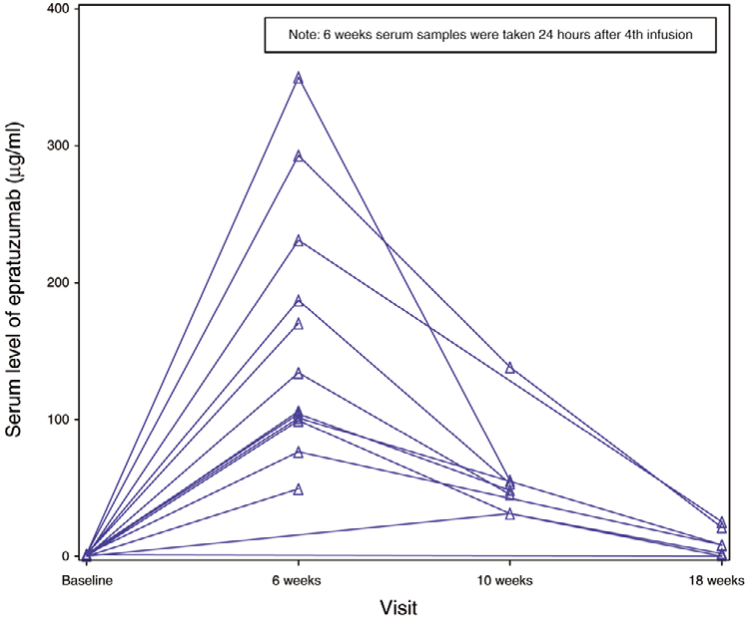
Serum levels of epratuzumab as detected by ELISA in the patients during the study.

Of the 14 patients, serum samples for analysis of pharmacokinetics and immunogenicity (HAHA) by ELISA assay were collected in a limited number of patients post-treatment at 6 weeks (*n *= 12), 10 weeks (*n *= 7) and 18 weeks (*n *= 7). Epratuzumab serum levels were measurable in all available samples through at least 10 weeks post-treatment and were still detectable above the 0.5 μg/ml assay limit in 5/7 samples evaluated at 18 weeks, with median values of 120 μg/ml (range 49 to 350) at 6 weeks, 48 μg/ml (range 31 to 138) at 10 weeks, and 8.3 μg/ml (range 1.82 to 25) at 18 weeks. Figure 4 shows the individual measurements over time. There was a single sample showing 1.42 μg/ml at 32 weeks. HAHA analysis gave no evidence of immunogenicity, with all post-treatment values either remaining below the 50 ng/ml sensitivity of the assay or not increased from baseline values prior to treatment.

### Immunology laboratory tests

**Figure 5:**
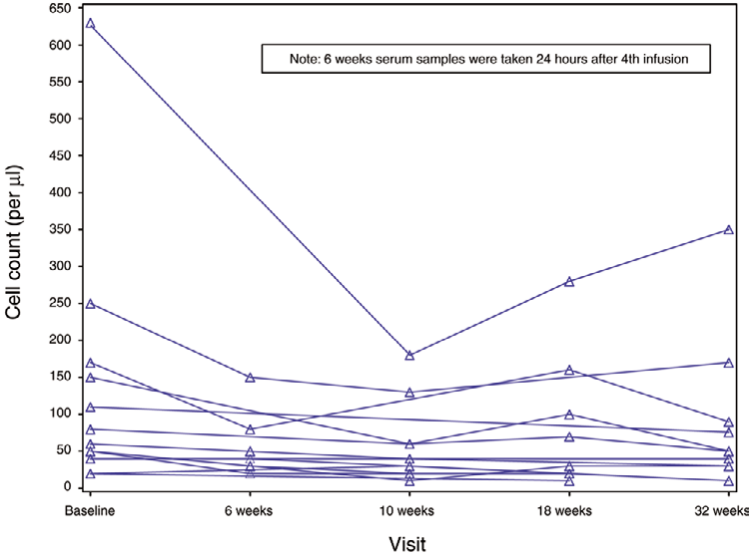
Follow-up of peripheral B cell levels during the study among individual study patients.

Table 7 shows that at the first evaluation after treatment, mean B cell levels decreased by 35% and persisted at these levels on subsequent evaluations (Figure 5), with no evidence of onset of recovery by the final study evaluation at 32 weeks (6 months post-treatment). In contrast, there does not appear to be any consistent pattern of decreases/increases in T cell levels or serum levels of IgG, IgA, or IgM following treatment (Table 7).

Although all 14 patients had measurable ANA titers (1:80 to 1:5,120) at study entry, no patient had consistent post-treatment decreases, including evaluations at 32 weeks (6 months post-treatment) follow-up (8 patients had no changes at any evaluation, 5 doubled their baseline titers at one or more evaluations, and one patient had an isolated decrease at one evaluation). Five patients had elevated anti-dsDNA antibodies (10 to 123 U/ml) at study entry, but none had any decreased post-treatment values (2 patients had no significant changes, and 3 had increases at one or more evaluations). C3 levels that were decreased or borderline for 3 patients at study entry remained virtually unchanged post-treatment, as did mean C3 values for all patients.

## Discussion

The pathogenesis of SLE remains enigmatic, but a central feature of this disease is the loss of immune tolerance and enhanced B cell activity. Although the number of B cells in the peripheral blood is often decreased, those that are present show characteristic alterations and have abnormal phenotypes indicative of activation [5,47]. Therefore, B cell depletion is an attractive therapeutic strategy for patients with SLE. The availability of the chimeric anti-CD20 antibody rituximab (Rituxan^® ^Genentech, South San Francisco, CA, USA; Biogen Idec, Boston, MA, USA) made it possible to test this hypothesis.

Initially, Isenberg and coworkers [19] treated 6 patients with active and otherwise refractory SLE (median BILAG score 14, range 9 to 27) with rituximab given in 500 mg doses 2 weeks apart with 2 doses of 750 mg iv cyclophosphamide and oral prednisolone cover (30 or 60 mg for 5 days). The treatment was safe and well tolerated, B cell depletion occurred, and BILAG total scores improved at 6 months (median 6, range 3 to 8). Looney and colleagues [6] initiated an open-label rituximab study of 17 patients with SLE (≥ 6 systemic lupus activity measurement, SLAM score) who were treated with either one 100 mg/m^2 ^dose, one 375 mg/m^2 ^dose, or four 375 mg/m^2 ^doses. Oral prednisone (40 mg for two doses) also was administered. B cell decreases were variable, with a 35% mean decrease persisting over the 6-month observation period, and clinical efficacy was demonstrated in patients with B cell depletion. Less than 6/17 of their patients developed human anti-chimeric antibody (HACA) at a level higher than or equal to 100 ng/ml when treated with this protocol.

All of these studies and case reports have so far been of short duration [7,48]. Usually, the B cell depletion in SLE is profound, as in patients with NHL, but shorter lasting. Therefore, it is very likely that cyclical therapy will be needed to provide long-term benefit for patients with SLE. While the immunogenicity of rituximab has not been clinically important (HACA < 1%) for the management of patients with NHL, approximately 4% of patients with rheumatoid arthritis developed HACA and 8% to 10% with SLE did so also, in spite of being treated with various doses of steroids and/or cytotoxic agents in combination with rituximab. Thus, a less immunogenic antibody (for example, a human or humanized form) is likely needed in the management of patients with autoimmune diseases, since it is expected that repeated dosing will be required in patients with such chronic diseases.

This initial study demonstrated that 360 mg/m^2 ^epratuzumab, a humanized CD22-specific monoclonal antibody, administered every other week for a total of 4 doses was safe and well-tolerated in SLE patients, with few significant adverse events, alterations of standard safety laboratory tests, and no evidence of immunogenicity. In addition to the minimal infusion reactions, the ability to complete an infusion within approximately 0.5 to 1 hour and the lack of immunogenicity are also likely to be more important treatment considerations in autoimmune diseases, as mentioned previously.

With this dosing schedule, virtually every patient with moderate disease activity (total BILAG score of 6 to 12) demonstrated symptomatic improvement using BILAG total scores. The BILAG total score results indicate that 77% of the patients achieved a ≥ 50% decrease in their overall disease activity at 6 weeks follow up. Furthermore, most patients (92%) continued to show reduced disease activity for at least 18 weeks, and even 38% showed a sustained response with BILAG reductions of 50% or more compared to study entry. Since this first study considered moderately active lupus patients with BILAG total scores of 6 to 12, the resulting heterogeneity precludes the identification of any preferential effect on one or the other BILAG domains as shown from different perspectives of efficacy analysis.

In addition to treating mild BILAG C-level symptoms, epratuzumab immunotherapy reduced all BILAG B-level activity in the majority of patients presenting with more serious disease, including patients with B-level activity in several body systems. The current data limit the conclusions that can be drawn regarding therapeutic effects for some systems, such as B-level disease in the neurological and renal systems, and only one case of lymphopenia in the hematological system showed improvement. In spite of small numbers, CD22-immunotherapy with epratuzumab appeared to be effective for treating disease in many of the other body/organ systems.

Although the biweekly dosing schedule used in this study demonstrated apparent activity, the serum levels of antibody measured here appear to be less than those in studies of NHL, where a weekly schedule of dose administrations has shown antitumor activity [42-44]. Hence, other dosing schedules in future clinical trials are warranted to assess the effects of increasing the serum levels of epratuzumab.

Compared to the complete depletion of B cells observed with rituximab, a long-lasting (at least 6 months, the last observation time) decrease of about 35% to 40% occurred with epratuzumab, with no apparent changes in T cells or immunoglobulin levels. As discussed earlier, the attractiveness of CD22 as a molecular target for therapy in SLE extends beyond the capability of epratuzumab to modestly decrease peripheral blood levels of B cells. CD22 is a cell surface receptor that is a member of the sialioadhesion family and an inhibitory co-receptor of BCR [34]. *In vitro *studies demonstrated that epratuzumab binding can induce CD22 phosphorylation [49] , and the current data from this study suggest that epratuzumab could potentially mediate direct pharmacological effects by negatively regulating certain hyperactive B cells. This hypothesis now needs to be tested. Interestingly, over the period of this study, patients clinically improved without clear evidence of reduction in ANA or anti-dsDNA titers. Similar observations have been reported with rituximab [19] , further supporting the hypothesis that targeted therapy impacting the hyperactive B cell compartment may be successful without needing to completely deplete the broader B cell population.

## Conclusion

This initial experience in lupus patients with mild to moderate symptoms demonstrated that 4 doses of 360 mg/m^2 ^epratuzumab immunotherapy are safe and well tolerated when infused within one hour, with consistent improvement observed in all patients for at least 12 weeks in the presence of modestly decreased (about 35%) peripheral B cell levels, and with no evidence of HAHA. Although this was an open-label study, consistent improvement was observed in all patients for at least 12 weeks, and there was reduction or elimination of disease activity across most body systems, regardless of the extent or the severity of the presenting disease activity. The duration of response was very heterogeneous for different BILAG domains, precluding firm conclusions at this time. As such, these results support conducting longer-term multicenter randomized controlled studies, which are now underway to examine the effects of epratuzumab in broader patient populations with autoimmune disease.

## Abbreviations

BCR = B cell antigen receptor; BILAG = British Isles Lupus Assessment Group; HACA = human anti-chimeric antibody; HAHA = human anti-human (epratuzumab) antibody; NCI CTC = National Cancer Institute Common Toxicity Criteria; NHL = non-Hodgkin lymphoma; SLE = systemic lupus erythematosus.

## Competing interests

TD, JK, and GRB declare research funding for this study provided by Immunomedics, Inc. WAW, NT, and DMG have employment and financial interests (stock) in Immunomedics, Inc., whichowns the antibody tested in this paper.

## Authors' contributions

All authors contributed to data interpretation and the final manuscript. TD and GRB were the principal investigators and were responsible for coordinating the study, while JK participated in patient selection and directed all patient related study procedures. DMG, TD and WAW designed the clinical trial protocol, and NT was responsible for data management and statistical analysis. TD and JK contributed equally to this work.

## Figures and Tables

**Table 1:** Number of patients with B-level disease activity at study entry in each BILAG body system

Body system	Number of patients	Contributing signs/symptoms* (number of patients)
I. General/constitutional	3	Fatigue/malaise/lethargy (3)
		Anorexia/nausea/vomiting (2)
		Unintentional weight loss > 5% (1)
II. Mucocutaneous	13	Malar erythema (11)
		Active localized discoid lesions (2)
		Mild maculopapular eruption (1)
III. Neurological	0	
IV. Musculoskeletal	2	Arthritis (2)
V. CV/Respiratory	2	Dyspnea (2)
		Pleuropericardial pain (2)
VI. Vasculitis	5	Minor cutaneous vasculitis (nailfold/digital vasculitis, purpura, urticaria) (5)
VII. Renal	0	
VIII. Hematology	1	Anemia (hemoglobin < 11 g/dL) (1)

*Signs and symptoms that contributed to the B-level disease activity according to BILAG rules.

**Table 2:** Number of patients with C-level disease activity at study entry in each BILAG body system

Body system	Number of patients	Contributing signs/symptoms* (number of patients)
I. General/Constitutional	11	Fatigue/malaise/lethargy (10)
		Anorexia/nausea/vomiting (1)
		Lymphadenopathy/splenomegaly (1)
		Pyrexia (documented) (1)
II. Mucocutaneous	1	Mild alopecia (1)
III. Neurological	10	Episodic migrainous headaches (8)
		Severe, unremitting headache (2)
IV Musculoskeletal	11	Arthralgia (10)
		Myalgia (9)
		Improving arthritis (1)
V. CV/Respiratory	2	Dyspnea (1)
		Pleuropericardial pain (1)
VI. Vasculitis	4	Raynaud's (3)
		Livido reticularis (1)
VII. Renal	4	Mild/stable proteinuria (4)
VIII. Hematology	11	Lymphocytopenia (< 1500 cells/μl) (10)
		Evidence of circulating anticoagulant (1)
		Decreased platelets (< 150,000/μl) (1)

*Signs and symptoms that contributed to the C-level disease activity according to BILAG rules.

**Table 3:** Number of patients with improvement from baseline BILAG B- and C-level activities

BILAG body system	6 weeks^a^	10 weeks	18 weeks
General (*N *= 14)^b^	6 (43%)	5 (36%)	2 (14%)
Mucocutaneous (*N *= 14)	11 (79%)	8 (57%)	6 (43%)
Neurological (*N *= 10)	7 (70%)	8 (80%)	6 (60%)
Musculoskeletal (*N *= 13)	9 (69%)	7 (54%)	4 (31%)
CV/Respiratory (*N *= 4)	3 (75%)	3 (75%)	3 (75%)
Vasculitis (*N *= 9)	4 (44%)	3 (33%)	3 (33%)
Renal (*N *= 4)	2 (50%)	1 (25%)	3 (75%)
Hematology (*N *= 12)	0 (0 %)	0 (0 %)	0 (0 %)
			
Overall^c ^(*N *= 14)	13 (93%)	14 (100%)	13 (93%)

^a^Twenty-four hours after fourth infusion. ^b^*N *= number of patients with involvement in a particular body system at entry. ^c^As applied to any BILAG body system.

**Table 4:** Number of patients with deteriorating BILAG activities from baseline

BILAG body system (*N *= 14)^a^	6 weeks^b^	10 weeks	18 weeks
General	0 (0 %)	0 (0 %)	0 (0 %)
Mucocutaneous	0 (0 %)	0 (0 %)	0 (0 %)
Neurological	0 (0 %)	0 (0 %)	0 (0 %)
Musculoskeletal	0 (0 %)	0 (0 %)	0 (0 %)
CV/Respiratory	0 (0 %)	0 (0 %)	1 (7 %)
Vasculitis	0 (0 %)	0 (0 %)	0 (0 %)
Renal	0 (0 %)	1 (7 %)	0 (0 %)
Hematology	1 (7 %)	1 (7 %)	2 (14%)
			
Overall^c^	1 (7 %)	2 (14%)	3 (21%)

^a^*N *= total number of patients. ^b^Twenty-four hours after fourth infusion. ^c^As applied to any BILAG body system.

**Table 5:** Number of patients in each BILAG body system with resolution of baseline B- and C-level disease activities

Body system	B level	C level
General	3/3 (100%)	0/11 (0%)
Mucocutaneous	4/13 (31%)	0/1 (0%)
Neurological	0/0	2/10 (20%)
Musculoskeletal	1/2 (50%)	1/11 (9%)
CV/Respiratory	0/2 (0%)	2/2 (100%)
Vasculitis	2/5 (40%)	0/4 (0%)
Renal	0/0	2/4 (50%)
Hematology	0/1 (0%)	0/11 (0%)

Resolution is defined as post-treatment improvement of baseline disease activity level by at least one category level (B to C, D, or E; C to D or E) at one or more evaluations up to 18 weeks, with no categorical deterioration from the baseline activity level prior to improvement, and no reversion to the baseline activity level once any improvement has occurred. Additionally note that 3 patients with multiple BILAG B involvement at baseline had completely resolved all B-level disease activities by 18 weeks.

**Table 6:** Post-treatment increases in NCI CTC v3.0 toxicity grades from baseline values

Labparameter	No increase	Toxicity increase	
		1 grade	2–3 grades
Hematology			
Hemoglobin	10	4	0
Platelets	12	2	0
WBC	11	3	0
ALC	6	6	2
ANC	13	1	0
Chemistry			
Creatinine	10	4	0
Total Bilirubin	14	0	0
Alkaline phosphatase	12	2	0
ALT (SGPT)	9	5	0
AST (SGOT)	10	4	0
GGT	12	2	0

ALC, absolute lymphocyte count, ANC, absolute neutrophil count, ALT, alanine aminotransferase, AST, aspartate aminotransferase, GGT, gamma glutamyl transferase, WBC, white blood cell

**Table 7:** Post-treatment changes of lymphocytes and immunoglobulins

	Baseline values and post-treatment percent change from baseline (mean ± SD)				
	Baseline	6 weeks	10 weeks	18 weeks	32 weeks
Lymphocytes	*N *= 14	*N *= 6	*N *= 8	*N *= 9	*N *= 11
B cells	123 ± 160 cells/μl	-35% ± 23%	-41% ± 41%	-34% ± 23%	-44% ± 21%
T cells	744 ± 554 cells/μl	+16% ± 80%	+28% ± 78%	+47% ± 109%	+17% ± 69%
Immunoglobulins		*N *= 12	*N *= 14	*N *= 10	*N *= 11
IgG	1,252 ± 355 mg/dl	+3% ± 8%	+5% ± 13%	+5% ± 9%	1% ± 13%
IgA	226 ± 94 mg/dl	+3% ± 11%	+8 ± 13%	+5% ± 12%	+10% ± 20%
IgM	117 ± 73 mg/dl	-12% ± 18%	-1% ± 23%	-6% ± 19%	-9% ± 9%

SD, standard deviation.
